# CASTOR1 phosphorylation predicts poor survival in male patients with *KRAS*-mutated lung adenocarcinoma

**DOI:** 10.1186/s13578-024-01307-4

**Published:** 2024-10-09

**Authors:** Suet Kee Loo, Gabriel Sica, Xian Wang, Tingting Li, Luping Chen, Autumn Gaither-Davis, Yufei Huang, Timothy F. Burns, Laura P. Stabile, Shou-Jiang Gao

**Affiliations:** 1 Cancer Virology Program, UPMC Hillman Cancer Center, Pittsburgh, PA USA; 2 Department of Microbiology and Molecular Genetics, University of Pittsburgh School of Medicine, Pittsburgh, PA USA; 3 Department of Pathology, University of Pittsburgh School of Medicine, Pittsburgh, PA USA; 4 UPMC Presbyterian Hospital, University of Pittsburgh Medical Center, Pittsburgh, PA USA; 5 Department of Medicine, University of Pittsburgh School of Medicine, Pittsburgh, PA USA; 6 Department of Electrical and Computer Engineering, Swanson School of Engineering, University of Pittsburgh, Pittsburgh, PA USA; 7 Cancer Biology Program, UPMC Hillman Cancer Center, Pittsburgh, PA USA; 8 Department of Pharmacology and Chemical Biology, University of Pittsburgh School of Medicine, Pittsburgh, PA USA

**Keywords:** Lung adenocarcinoma, LUAD, Biomarker, Cytosolic arginine sensor for mTORC1 subunit 1, CASTOR1, Mammalian target of rapamycin complex 1, mTORC1, *KRAS*

## Abstract

**Background:**

Lung cancer, a leading global cause of cancer-related mortality, necessitates enhanced prognostic markers for improved treatment outcomes. We have previously shown a tumor suppressive role of cytosolic arginine sensor for mTORC1 subunit 1 (CASTOR1), which is targeted for degradation upon phosphorylation at S14 (pCASTOR1) in multiple types of cancer. This study focuses on the predictive value of pCASTOR1 in lung adenocarcinoma (LUAD) patients with *KRAS* mutations.

**Results:**

Employing a newly developed pCASTOR1 specific antibody, we found that tumor cells exhibited significantly elevated pCASTOR1 scores compared to non-tumor cells (*P* < 0.05). Higher pCASTOR1 scores predicted poorer overall survival (OS) (HR = 3.3, *P* = 0.0008) and relapse-free survival (RFS) (HR = 3.0, *P* = 0.0035) in male patients with *KRAS* mutations. pCASTOR1 remained an independent predictor for OS (HR = 4.1, *P* = 0.0047) and RFS (HR = 3.5, *P* = 0.0342) after controlling for other factors. Notably, in early-stage LUAD, elevated pCASTOR1 scores were associated with significantly worse OS (HR = 3.3, *P* = 0.0176) and RFS (HR = 3.1, *P* = 0.0277) in male patients with *KRAS* mutations, akin to late-stage patients.

**Conclusion:**

Elevated pCASTOR1 scores serve as biomarkers predicting poorer OS and RFS in male LUAD patients with *KRAS* mutations, offering potential clinical utility in optimizing treatment strategies for this subgroup.

**Supplementary Information:**

The online version contains supplementary material available at 10.1186/s13578-024-01307-4.

## Introduction

Lung cancer stands as the leading cause of cancer-related fatalities worldwide, affecting both men and women [1]. The two primary subtypes, non-small cell lung carcinoma (NSCLC) and small-cell lung carcinoma (SCLC), collectively constitute 85% and 15% of lung cancer cases, respectively [2, 3]. Among NSCLC cases, lung adenocarcinoma (LUAD) comprises 50%, with the remaining subtypes including squamous cell carcinoma, large cell carcinoma, and some undefined variants [4, 5].

NSCLC exhibits a notable sex-based distribution, with LUAD more frequently diagnosed in women, while men are more commonly affected by squamous cell carcinoma [6]. Approximately 30% of LUAD cases harbor Kirsten rat sarcoma (*KRAS*) mutations, often linked to cigarette smoking [7, 8]. *KRAS* mutations typically manifest as single-base missense mutations, predominantly occurring in codon 12 of exon 2, with lesser occurrences at codons 13 and 61 [9]. The G12C single-base missense mutation stands out as the most prevalent *KRAS* mutation in LUAD, accounting for 13% of cases [10]. In contrast to *KRAS* mutations, epidermal growth factor receptor (*EGFR*) mutations are more prevalent among nonsmokers. *EGFR* mutations are identified in 15% of LUAD cases and commonly exhibit mutual exclusivity with *KRAS* mutations [11, 12]. This distinction underscores the diverse molecular landscape of LUAD, influenced by both smoking history and specific genetic alterations.

Over the preceding decades, the treatment landscape for lung cancer has undergone a transformative shift, transitioning from conventional chemotherapy to molecular targeted therapy and immunotherapy. This evolution has substantially enhanced the prognosis for patients afflicted with the disease [5]. Despite these advancements, a notable proportion of patients either exhibit resistance to treatment or encounter disease progression and recurrence [5]. Similar to many other cancer types, LUAD encompasses a diverse array of *KRAS* mutations and other genetic alterations, underscoring the imperative for personalized therapeutic strategies tailored to specific genotypes [13]. Recognizing this heterogeneity, there has been a concerted effort to implement highly effective therapies specifically designed for distinct disease subtypes and stages, with particular emphasis on early-stage management [14–17]. This nuanced approach aims to optimize treatment outcomes by addressing the unique genetic profiles of individual patients, marking a crucial step towards achieving precision medicine in the context of lung cancer therapeutics.

The mammalian target of rapamycin complex 1 (mTORC1) is a pivotal regulator orchestrating diverse cellular functions, ranging from cell proliferation to survival. Its frequent activation in cancers, including LUAD, underscores its significance in tumorigenesis [18–20]. The dysregulation of the mTORC1 pathway is a common feature across various cancer types, emphasizing its role as a key player in oncogenesis [18]. Indeed, inhibitors targeting mTORC1 have demonstrated remarkable efficacy against numerous cancer types, highlighting the therapeutic potential of modulating this pathway [18]. The intricate regulation of mTORC1 involves complex interplay with nutrient and growth factor pathways, contributing to its multifaceted impact on cellular processes [18]. Understanding the specific pathways that mediate mTORC1 activity in distinct tumors, particularly LUAD, holds substantial promise in tailoring optimal treatment regimens. Moreover, such insights into mTORC1 regulation could pave the way for the development of effective prognostic markers, facilitating more precise prognostication and clinical management of LUAD.

The cytosolic arginine sensor for mTORC1 subunit 1 (CASTOR1) functions as a negative regulator of mTORC1. In its dimerized state, CASTOR1 inhibits the GATOR2 complex by binding to Mios, leading to the suppression of mTORC1 activity [19, 20]. Upon sensing arginine, CASTOR1 dissociates from the GATOR2 complex, resulting in the activation of mTORC1 [19, 20]. Our previous works have established a tumor-suppressive role of CASTOR1, demonstrating that lower levels of CASTOR1 expression are associated with worse survival across at least 10 types of cancer [21, 22]. Overexpression of CASTOR1 has been shown to attenuate mTORC1 activation in KSHV-transformed cells and breast cancer cells, thereby suppressing tumorigenesis [21, 22]. Since the tumor microenvironment often exhibits low arginine levels, cancer cells have evolved specific mechanisms to inhibit CASTOR1 function [23]. In KSHV-transformed cells, the virus encodes two specific miRNAs to inhibit CASTOR1 expression [22]. In other cancer types, CASTOR1 is phosphorylated at S14 (pCASTOR1) by AKT, sensitizing it to ubiquitination by the RNF167 E3 ligase and subsequent proteasome-mediated degradation [21]. Given that AKT is activated by various growth factors and mutations in genes regulating AKT in cancer, CASTOR1 serves as a crucial integrator of signals from both nutrients and growth factors, thereby modulating mTORC1 activity, cell proliferation, and survival [23].

In this study, we explore the potential of phosphorylated CASTOR1 at S14 (pCASTOR1) as a prognostic marker in LUAD. Using a site-specific antibody targeting pCASTOR1, we examined LUAD tumors and adjacent non-tumor tissues in tissue microarrays (TMAs). Our observations revealed that tumor cells exhibit significantly elevated pCASTOR1 levels compared to non-tumor cells. To evaluate the prognostic value of pCASTOR1, we analyzed its association with overall survival (OS) and recurrence-free survival (RFS) in LUAD patients. Our findings indicate that pCASTOR1 scores serve as predictors of survival outcomes specifically in male LUAD patients with *KRAS* mutations. Higher pCASTOR1 scores were found to significantly correlate with worse OS and RFS in these patients. This association was particularly pronounced in early-stage (stage I-II) *KRAS* mutant LUAD male patients, where elevated pCASTOR1 scores conferred significantly worse OS and RFS, akin to outcomes observed in stage III-IV cases. These results pinpoint a subgroup of early-stage patients with *KRAS* mutations who are at a heightened risk of developing recurrent disease. Given that treatments for early-stage patients typically involve surgery, radiotherapy, and chemotherapy, this identified patient group may benefit substantially from more tailored and specific treatment approaches. The implications of our findings underscore the potential for pCASTOR1 as a valuable biomarker in guiding treatment for male LUAD patients with *KRAS* mutations, particularly for those with early-stage disease.

## Methods

### Study approval and population

This retrospective study was approved by the Institutional Review Board of the University of Pittsburgh (Protocol ID: STUDY22020142) and informed consent from patients were obtained in accordance with the Declaration of Helsinki. The study encompassed a cohort of patients diagnosed with LUAD who underwent thoracic surgical procedures at the University of Pittsburgh Medical Center (UPMC) (Table S1). The cohort consisted of 165 LUAD patients, each characterized by known *KRAS* or *EGFR* mutation status. TMAs were constructed as previously described, excluding cases exhausted of tissue cores, cases with insufficient cells for assessment of pCASTOR1 scores, cases with incomplete information for *EGFR* or *KRAS* genotyping, cases without *EGFR* fluorescent in-situ hybridization (FISH) ratio and cases that were both *KRAS* positive and having *EGFR* FISH ratio above 2.0 [24].

Demographic and clinical data, encompassing variables such as age, sex, race, stage, smoking status, and outcomes, were sourced from the UPMC Cancer Registry. OS was calculated from the date of disease diagnosis to the date of the last follow-up or the date of death. RFS was determined from the date of diagnosis to the date of recurrence, death, or the last follow-up, with recurrence or death considered as events. Cases lacking information on specific variables, such as stage, OS, RFS or pCASTOR1 scores were excluded from the corresponding analyses involving those variables.

### Immunohistochemistry and evaluation

Immunochemistry (IHC) staining was performed using a site-specific antibody targeting phosphorylated CASTOR1 at S14 (pCASTOR1). The rabbit antibody was commercially generated by Cocalica Biologicals (Stevens, PA) through immunization with a KLH-conjugated phosphopeptide C-EHRVRVL[pS]VARP and subsequently affinity-purified using a column generated with the unconjugated phosphopeptide. Following deparaffinization, antigen retrieval was performed by heating the slide in citrate buffer at pH 6.0 with a microwave oven at full power for 20 min. Quenching of endogenous peroxidase was performed with 3% hydrogen peroxide followed by blocking with 5% bovine serum albumin (BSA). Incubation of the primary antibody was performed at 4 °C overnight at a 1:50 dilution. Secondary antibody incubation was performed at room temperature for 1 h. A specific signal was detected using horseradish peroxidase (HRP)-based diaminobenzidine (DAB) as a substrate and hematoxylin as the counterstain.

After the completion of IHC staining, a pathologist blinded to the cases scored the tissues for both intensities and frequencies of positive cells at different pCASTOR1 levels in the tumors and adjacent non-tumor cells, resulting in two separate sets of scores: one for tumor cells and another for non-tumor cells. Staining intensity was scored using a 4-tier system as follows: 0 for no staining, and 1, 2, and 3 for weak, moderate, and strong staining, respectively. Staining frequency was scored in 5% increment from 0 to 100% for each staining intensity with total score of 100%. Final scores for both the tumor and non-tumor cells were obtained by averaging the scores of all the replicate cores from the same patients. The pCASTOR1 scores used in all the analyses were obtained by summing scores of moderate and strong staining intensities. Cores with insufficient cells for assessing pCASTOR1 level in tumor or non-tumor cells were not scored and excluded from analysis. The obtained scores were then subjected to analysis to explore their association with survival outcomes. OS and RFS were assessed at every quartile of pCASTOR1 score. This iterative approach aimed to identify the most significant cut-off point that could effectively stratify patients into two groups, i.e. those with low and high pCASTOR1 scores, yielding distinct survival outcomes.

### Western-blotting

Western-blotting was performed as previously described [25]. Total proteins from cells were prepared in Laemmli Buffer supplemented with a cocktail of protease inhibitors (Roche Diagnostics). Proteins separated by SDS-PAGE were transferred onto a nitrocellulose membrane (Invitrogen). The membranes were blocked for 1 h and incubated overnight at 4 °C with rabbit polyclonal antibodies to CASTOR1 (1:1000) or pCASTOR1 (1:1000), or a rabbit monoclonal antibody to GAPDH (1:1000, Cell Signaling Technology, #5174). Membranes were then incubated with an HRP-conjugated secondary antibody for 1 h at room temperature. The membranes were then rinsed with TBST buffer and soaked in the SuperSignal West Femto Substrate (Thermo Scientific, #24096). Signals were visualized by the ChemiDoc Imaging System and quantified using Image Lab software (Bio-Rad).

### Statistical analysis

The univariate OS and RFS analyses were conducted using the Kaplan-Meier method. The comparison of survival between groups was accomplished using the log-rank test, executed with GraphPad (Version 10.1.0). To ascertain multivariate survival significance and hazard ratios, Cox regression analysis was employed (IBM SPSS Statistics, Version 28.0.1.1). For comparisons of pCASTOR1 scores between tumor and non-tumor cells, paired t-test was utilized. All statistical analyses adhered to a significance threshold of *P* < 0.05.

## Results

### Patient characteristics

The study consisted of 165 LUAD patients with characteristics presented in Table S1. The median age of the patients was 66.0 years, 74 (44.8%) were male, 153 (92.7%) were white, and most of them were former smokers or nonsmokers with only 42 (25.5%) smokers at diagnosis. *KRAS* mutations were present in 76 (46.1%) patients and *EGFR* mutations were present in 43 (26.1%) patients. Overall median RFS was 58 months (95% CI: 40–78 months) and overall median OS was 76 months (95% CI: 60–102 months).

### Lung adenocarcinoma tumors have more pCASTOR1-positive tumor cells than non-tumor cells 

**Fig. 1:**
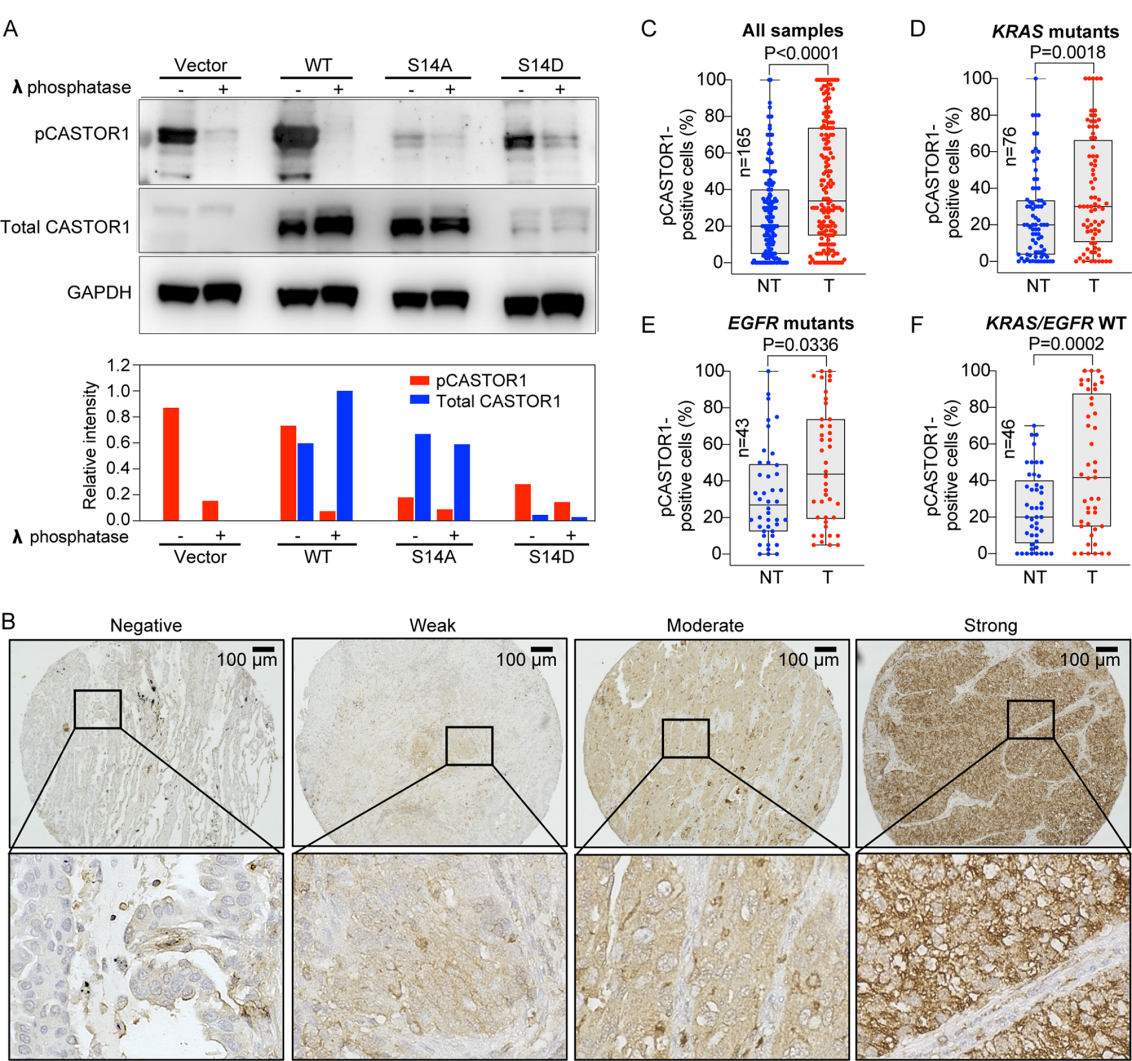
Detection of pCASTOR1 in LUAD tumor and non-tumor cells. **A** pCASTOR1 antibody specifically detects phosphorylated CASTOR1 at S14 site. Untreated and λ-phosphatase-treated proteins from 293T cells transfected with vector control, wild-type (WT) CASTOR1, CASTOR1 with a phosphorylation dead mutation at S14 by mutating serine to alanine (S14A) or constitutively phosphorylation mimic at S14 by mutating serine to aspartic acid (S14D) were examined with an antibody generated with a CASTOR1 S14 phosphopeptide. Band intensities of total CASTOR1 and pCASTOR1 were quantified using Image Lab software (Bio-Rad) and normalized against those of GAPDH. **B** pCASTOR1 has different staining intensities in LUAD tumors. Representative pCASTOR1 images of LUAD tumors showing tumor with absence of pCASTOR1 staining and tumors stained with weak, moderate or strong intensity of pCASTOR1. Images on the lower panel correspond to the digitally enlarged boxed region on the top panel. **C-F** Comparison of pCASTOR1 scores between tumor cells (T) and non-tumor cells (NT) in all patients (*n* = 165) (**C**), patients with *KRAS* mutations (*n* = 76) (**D**), patients with *EGFR* mutations (*n* = 43) (**E**) and patients without *KRAS* or *EGFR* mutations (*n* = 46) (**F**). Cores with insufficient cells for assessment of pCASTOR1 scores in tumor or non-tumor cells were excluded from the analysis. P-values were assessed with two-tailed paired t-test

We generated an antibody targeting the phosphorylated S14 site of CASTOR1 (pCASTOR1) using a phosphopeptide. To validate the specificity of the antibody, we examined 293T cells overexpressing wild-type (WT) CASTOR1, CASTOR1 with a constitutively phosphorylation mimic at S14 by mutating the serine to aspartic acid (S14D) or CASTOR1 with a phosphorylation dead mutation at S14 by mutating the serine to alanine (S14A) [21]. The antibody detected robust endogenous pCASTOR1 signal in cells expressing the vector control, which was significantly reduced upon treatment with λ-phosphatase (Fig. 1A). CASTOR1 is targeted for degradation by RNF167 E3 ligase upon S14 phosphorylation [21]. Consequently, cells expressing S14D CASTOR1 had much lower total protein levels than those expressing WT or S14A CASTOR1 (Fig. 1A). As expected, the pCASTOR1/CASTOR1 ratio of S14D CASTOR1 was much higher than that of WT or S14A CASTOR1. Treatment with λ-phosphatase reduced pCASTOR1 signals in all types of cells. These results confirmed the specificity of the pCASTOR1 antibody.

Using the pCASTOR1 antibody, we then stained the LUAD tissue cores from 165 patients. The pCASTOR1 antibody stained tumor cells with variable intensities and frequencies while non-tumor cells had much lower intensities and frequencies (Fig. 1B-F). We compared pCASTOR1-positive cells between tumor cells and non-tumor cells. Tumor cells consistently had significantly higher numbers of pCASTOR1-positive cells than those of non-tumor cells consisting of stromal and immune cells (*P* < 0.05 for all cases in Fig. 1C). This trend persisted when cases were examined according to the status of *KRAS* or *EGFR* mutations (*KRAS* mutants in Fig. 1D, *n* = 76; *EGFR* mutants in Fig. 1E, *n* = 43; *KRAS* WT and *EGFR* WT in Fig. 1F, *n* = 46).

### pCASTOR1 scores predict survival in male LUAD patients with *KRAS* mutations

We investigated pCASTOR1 as a prognostic marker for LUAD patients. The examination of OS and RFS revealed several predictors, including sex, age at diagnosis and cancer stage (Fig. S1). These results were consistent with findings from other studies [26–28]. While cigarette smoking was reported to be a risk factor for *KRAS* mutant lung cancer [29], there was no association of OS and RFS with smoking in this cohort. This could be due to the fact that patients from this cohort were predominantly former smokers and nonsmokers.

**Fig. 2:**
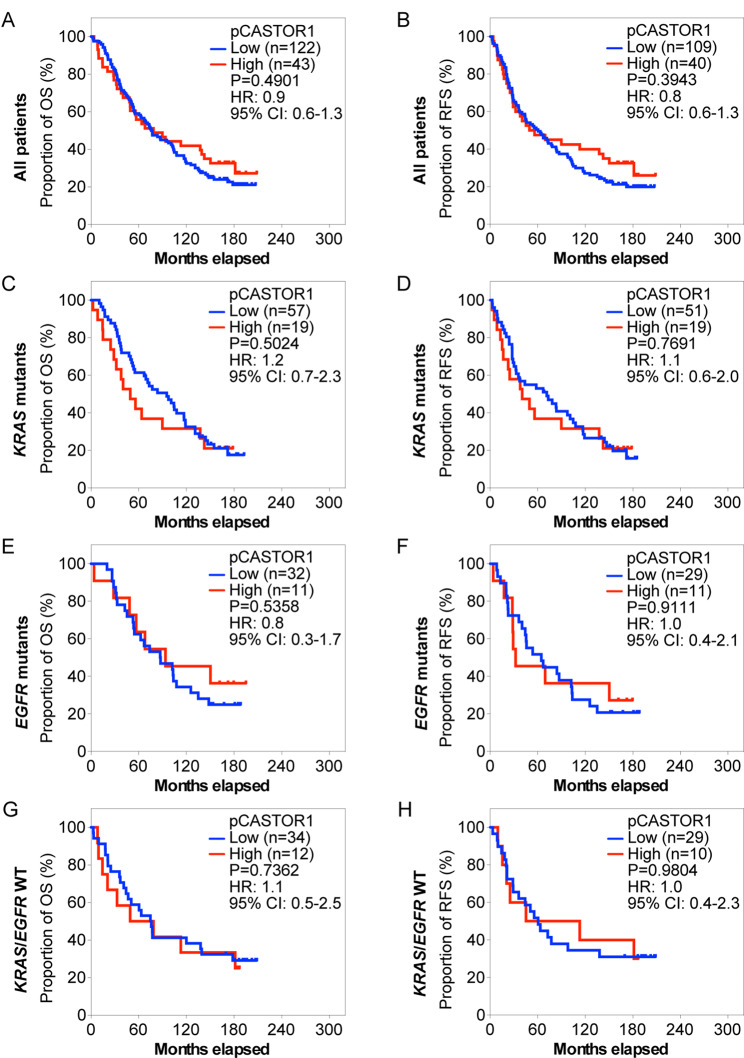
OS and RFS of LUAD patients stratified according to pCASTOR1 scores. **A-H** All LUAD patients (**A** and **B**), patients with *KRAS* mutations (**C** and **D**), patients with *EGFR* mutations (**E** and **F**), and patients without any *KRAS* or *EGFR* mutations (**G** and **H**) were analyzed for OS (**A**, **C**, **E** and **G**) and RFS (**B**, **D**, **F** and **H**). X-axis: months elapsed. Y-axis: proportion of OS (%) or RFS (%). HR: Hazard ratio; CI: Confidence interval

**Fig. 3:**
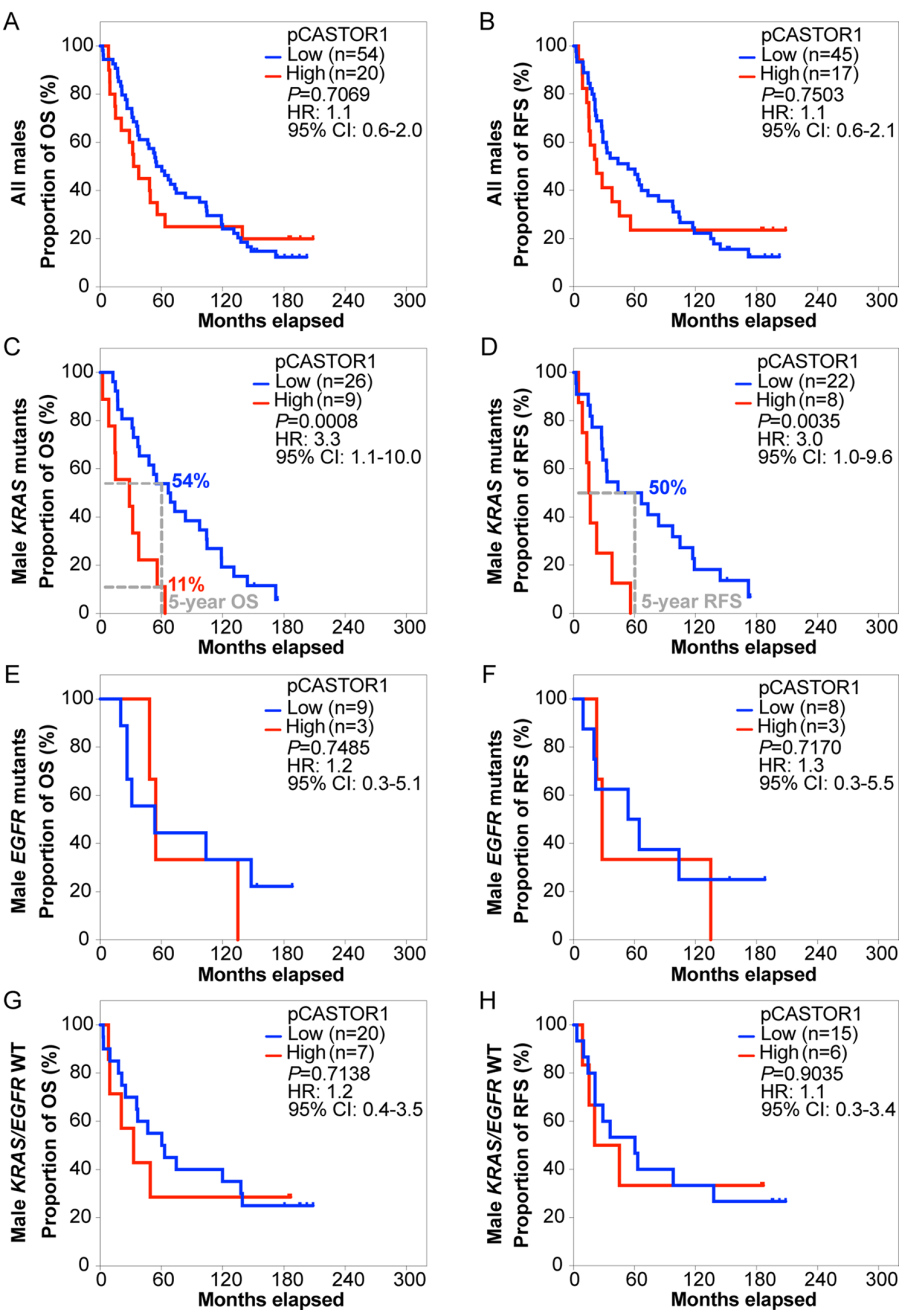
OS and RFS of LUAD male patients grouped according to pCASTOR1 scores. **A-H** All male patients (**A** and **B**), male patients with *KRAS* mutations (**C** and **D**), male patients with *EGFR* mutations (**E** and **F**), and male patients without any *KRAS* or *EGFR* mutations (**G** and **H**) were analyzed for OS (**A**, **C**, **E** and **G**) and RFS (**B**, **D**, **F** and **H**). X-axis: months elapsed. Y-axis: proportion of OS (%) or RFS (%), HR: Hazard ratio; CI: Confidence interval

**Fig. 4:**
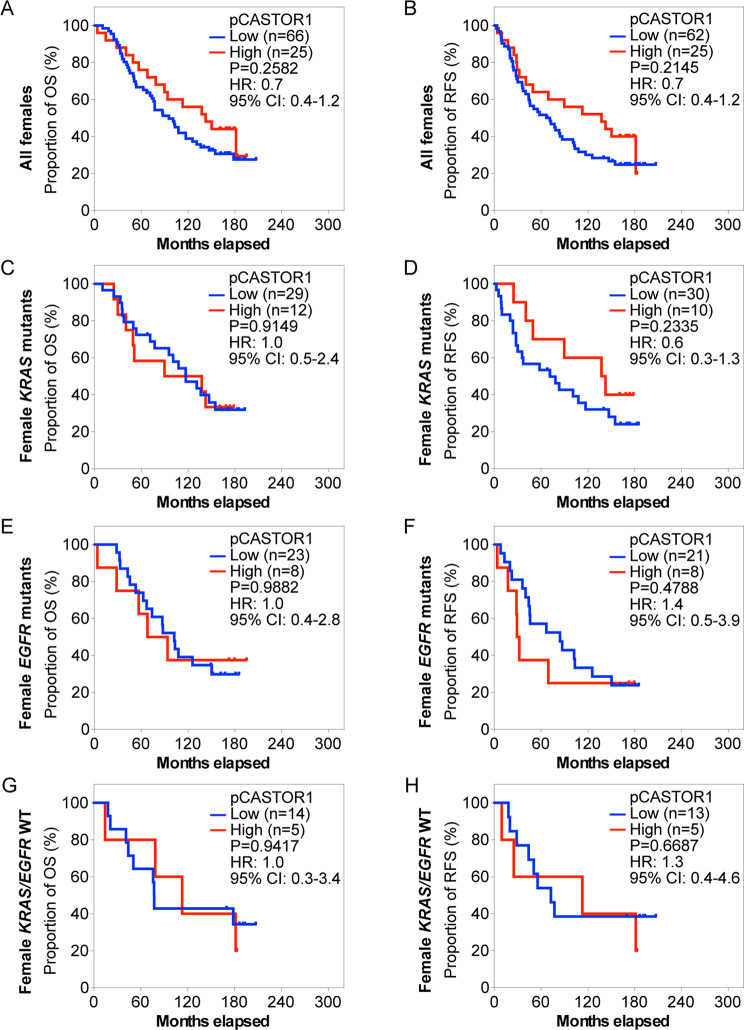
OS and RFS of female LUAD patients stratified according to pCASTOR1 scores. **A-H** All female LUAD patients (**A** and **B**), patients with *KRAS* mutations (**C** and **D**), patients with *EGFR* mutations (**E** and **F**), and patients without any *KRAS* or *EGFR* mutations (**G** and **H**) stratified according to pCASTOR1 scores for OS (**A**, **C**, **E** and **G**) and RFS (**B**, **D**, **F** and **H**). X-axis: months elapsed. Y-axis: proportion of OS (%) or RFS (%). HR: Hazard ratio; CI: Confidence interval

Next, we stratified the patients based on pCASTOR1 scores. However, no significant differences in survival outcomes were observed in the entire cohort or when stratified by *KRAS* or *EGFR* mutant genotype (Fig. 2A-H, Table S2). Previous studies have implicated sex hormones in the development and therapeutic outcomes of lung cancer [6]. Indeed, female patients had significantly better OS and RFS than male patients (HR = 1.8, *P* = 0.0006 for OS and HR = 1.5, *P* = 0.0290 for RFS, Fig. S1). This trend persisted in patients harboring *KRAS* mutations (HR = 2.4, *P* = 0.0004 for OS and HR = 1.9, *P* = 0.0108 for RFS) but not those with *EGFR* mutations or without any *KRAS* and *EGFR* mutations (Fig. S1). When patients were stratified according to pCASTOR1 scores in tumor cells, no differences were observed in OS and RFS between patients with low vs. high pCASTOR1 scores in male (Fig. 3A-B) or female patients (Fig. 4A-B).

Because of the different prevalence of *KRAS* and *EGFR* mutations in male and female LUAD patients [30, 31], we investigated the survival differences among male and female patients by genotype. Among male patients with *KRAS* mutations, those with high pCASTOR1 scores had significantly worse OS (HR = 3.3, *P* = 0.0008) and RFS (HR = 3.0, *P* = 0.0035) compared to those with low pCASTOR1 scores (Fig. 3C-D). All patients with high pCASTOR1 scores experienced events of recurrence within 5 years, and only 11% of them achieved 5-year OS with none surviving past 10 years. In contrast, among patients with low pCASTOR1 scores, 50% of them had 5-year OS and RFS, and 20% achieved 10-year OS and RFS (Fig. 3C-D). pCASTOR1 score remained significantly predictive of both OS (HR = 4.1, *P* = 0.0047) and RFS (HR = 3.5, *P* = 0.0342) in male *KRAS* mutant LUAD patients after controlling for age at diagnosis, stage and smoking history (Table 1). Conversely, there was no association of pCASTOR1 scores with outcomes in female *KRAS* mutant LUAD patients (Fig. 4C-D, Table S3). pCASTOR1 score was not predictive of OS or RFS in male or female *EGFR* mutant LUAD or *EGFR*/*KRAS* WT patients (Figs. 3E-H and 4E-H).

**Table 1:** Multivariate analysis of male patients with *KRAS* mutations

Characteristics	OS				RFS			
	*n* (%)	HR	95.0% CI	*P*-value	*n* (%)	HR	95.0% CI	*P*-value
Age (year-old)								
< 65	11 (33.3)	1.0			8 (27.6)	1.0		
≥ 65	22 (66.7)	0.9	0.4–2.1	0.7503	21 (72.4)	1.8	0.7-5.0	0.2305
Stage								
I & II	23 (69.7)	1.0			22 (75.9)	1.0		
III & IV	10 (30.3)	5.0	1.8–13.6	0.0017	7 (24.1)	2.8	0.8–9.7	0.0951
Pack year								
< 40	13 (39.4)	1.0			12 (41.4)	1.0		
≥ 40	20 (60.6)	1.6	0.7–3.5	0.2678	17 (58.6)	0.9	0.4–2.1	0.8477
pCASTOR1								
Low	24 (72.7)	1.0			21 (72.4)	1.0		
High	9 (27.3)	4.1	1.5–11.0	0.0047	8 (27.6)	3.5	1.1–11.2	0.0342

HR: hazard ratio; CI: Confidence interval; OS: Overall survival; RFS: Recurrence-free survival; Pack year: total cigarette packs smoked in lifetime

### High pCASTOR1 scores confer worse survival in early-stage male LUAD patients with *KRAS* mutations

**Fig. 5:**
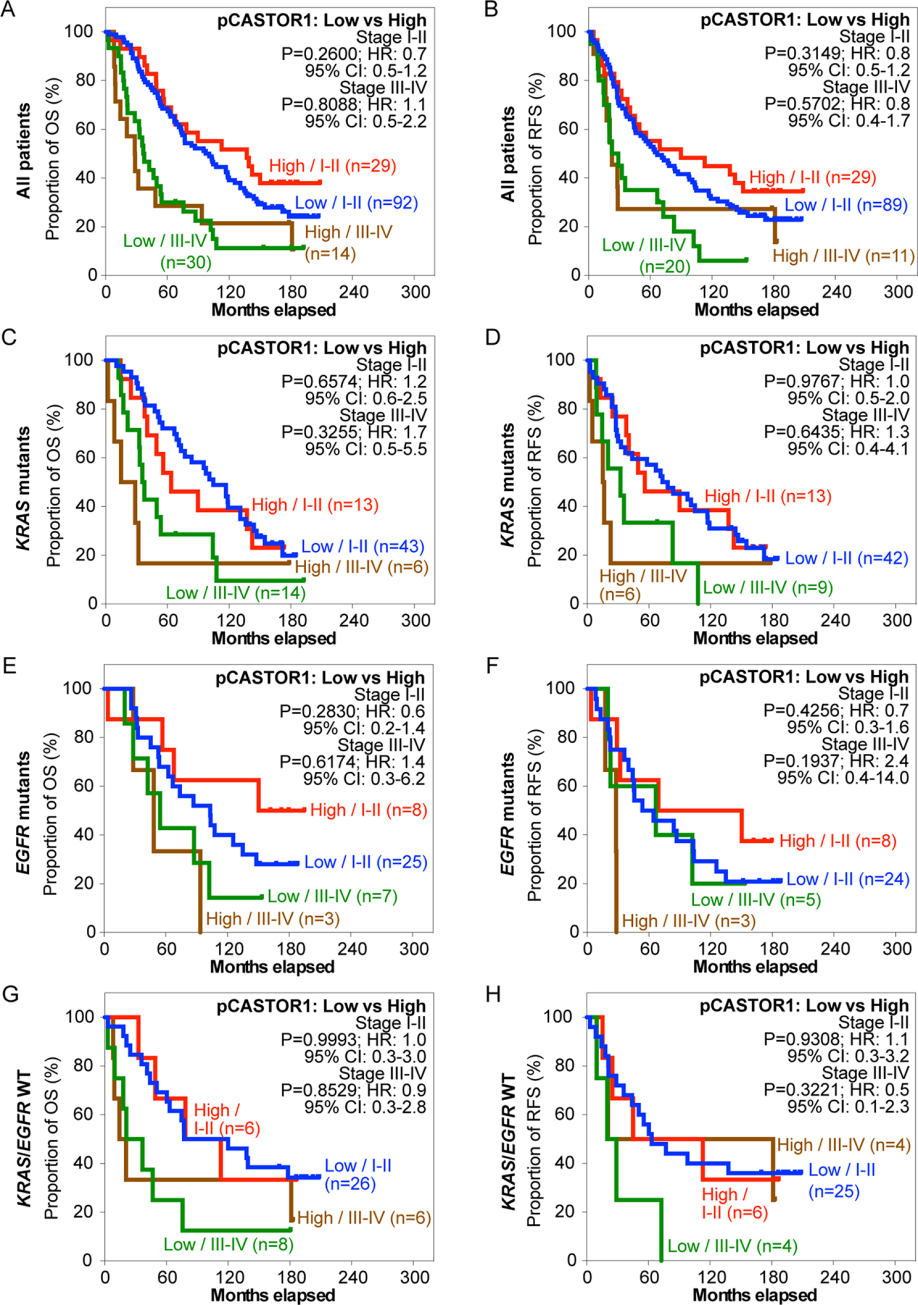
OS and RFS of LUAD patients stratified according to cancer stage and pCASTOR1 scores. **A-H** All patients (**A** and **B**), patients with *KRAS* mutations (**C** and **D**), patients with *EGFR* mutations (**E** and **F**), and patients without any *KRAS* or *EGFR* mutations (**G** and **H**) were analyzed for OS (**A**, **C**, **E** and **G**) and RFS (**B**, **D**, **F** and **H**). Early-stage patients were those in stages I and II while late-stage patients were those in stages III and IV. X-axis: months elapsed. Y-axis: proportion of OS (%) or RFS (%). HR: Hazard ratio; CI: Confidence interval

**Fig. 6:**
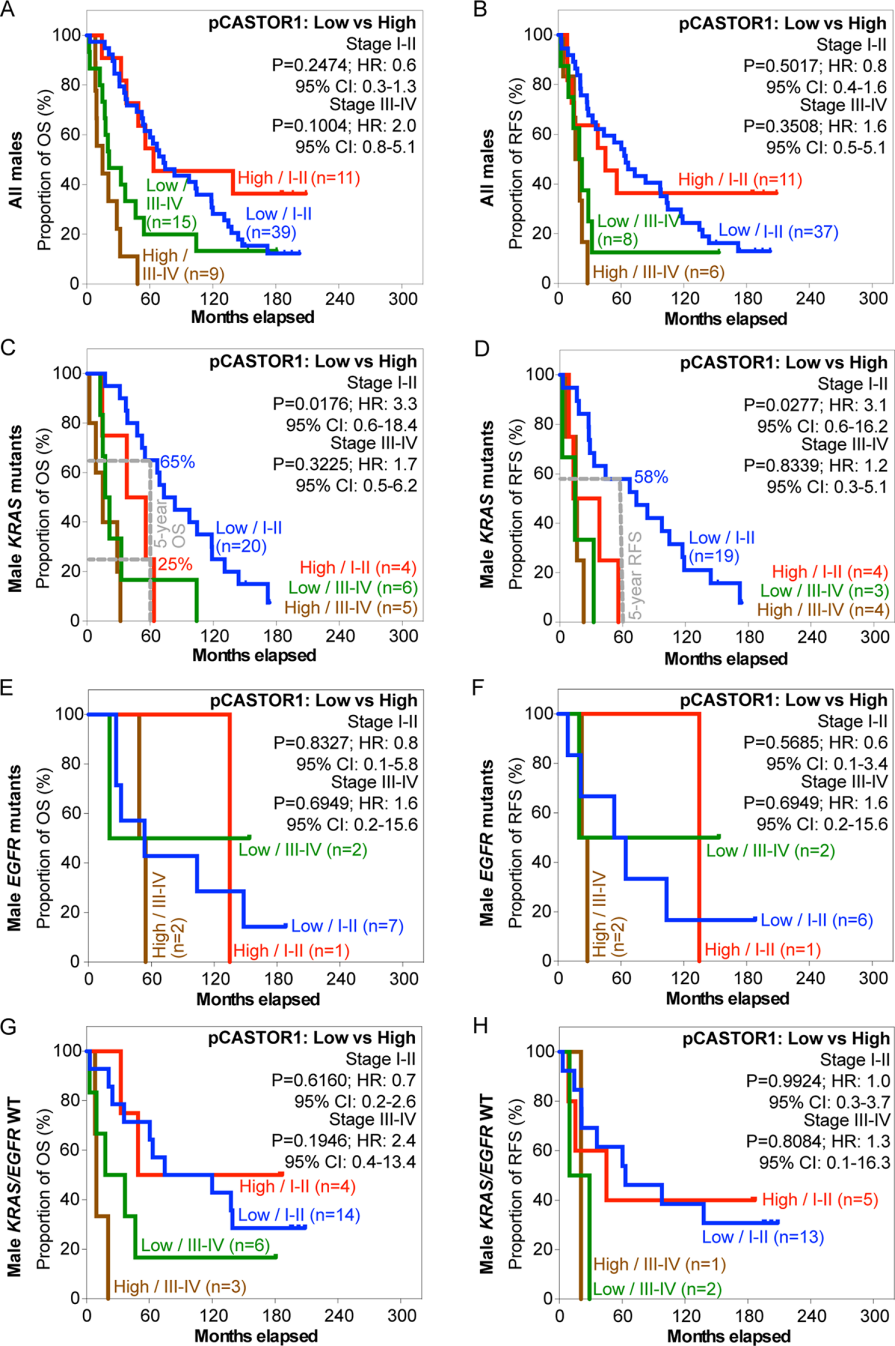
OS and RFS of LUAD male patients stratified according to cancer stage and pCASTOR1 scores. **A-H** All male patients (**A** and **B**), male patients with *KRAS* mutations (**C** and **D**), male patients with *EGFR* mutations (**E** and **F**), and male patients without any *KRAS* or *EGFR* mutations (**G** and **H**) were analyzed for OS (**A**, **C**, **E** and **G**) and RFS (**B**, **D**, **F** and **H**). Early-stage patients were those in stages I and II while late-stage patients were those in stages III and IV. X-axis: months elapsed. Y-axis: proportion of OS (%) or RFS (%). HR: Hazard ratio; CI: Confidence interval

**Fig. 7:**
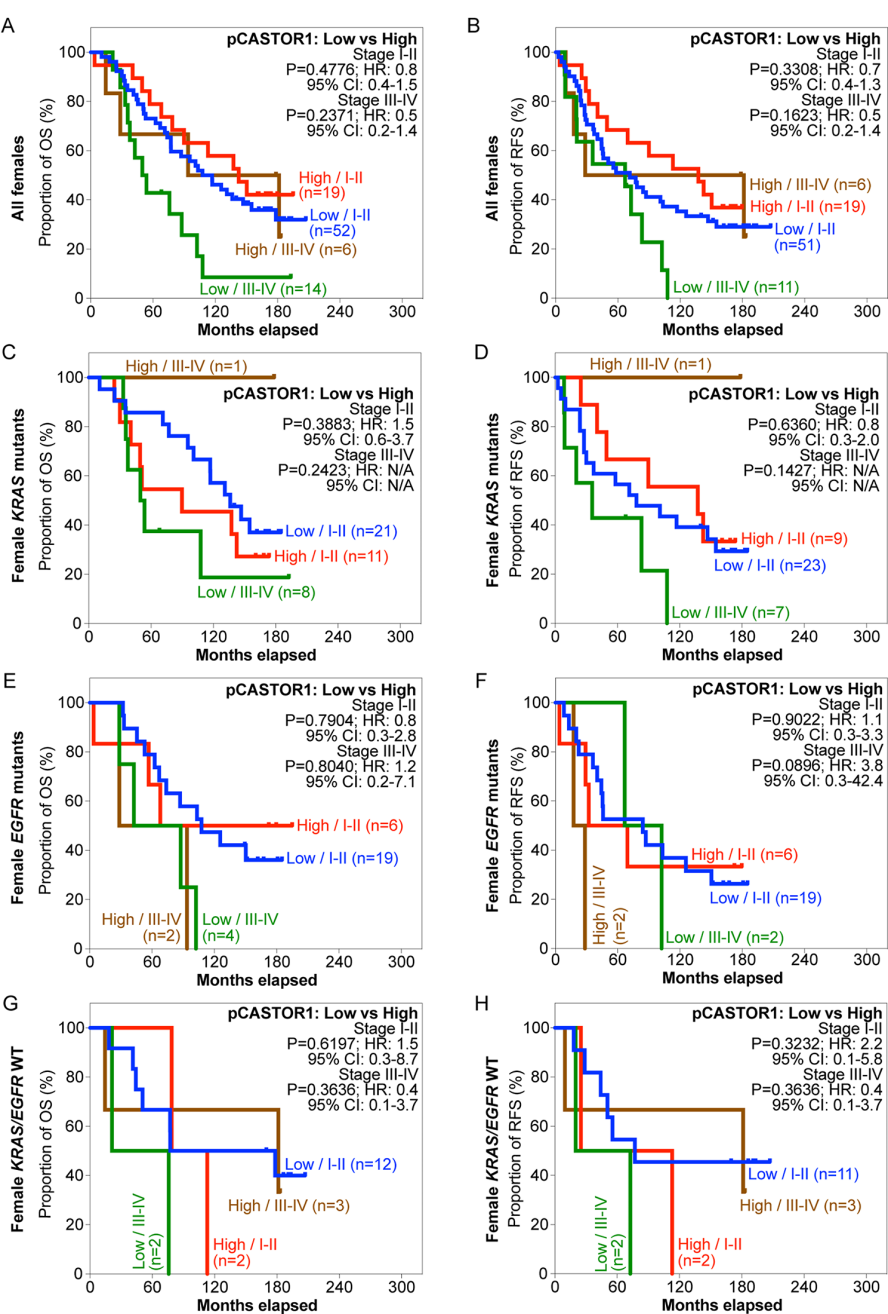
OS and RFS of female LUAD patients stratified according to cancer stage and pCASTOR1 scores. **A-H** All female LUAD patients (**A** and **B**), female patients with *KRAS* mutations (**C** and **D**), female patients with *EGFR* mutations (**E** and **F**), and female patients without any *KRAS* or *EGFR* mutations (G and H) were analyzed for OS (**A**, **C**, **E** and **G**) and RFS (**B**, **D**, **F** and **H**). X-axis: months elapsed. Y-axis: proportion of OS (%) or RFS (%). HR: Hazard ratio; CI: Confidence interval

We next stratified LUAD patients with early- (stage I-II) or late-stage (stage III-IV) disease based on pCASTOR1 scores. Cancer stage was predictive of survival, particularly in patients with *KRAS* mutations (Fig. S1). In general, patients in late-stage had almost twice the risk compared to those in early-stage to develop events of disease recurrence or death. In patients with *EGFR* mutations, and without either *KRAS* or *EGFR* mutations, cancer stage was only predictive of OS but not RFS (Fig. S1). In agreement with the results described above, when stratified by cancer stage, pCASTOR1 score was not a predictor in all patients regardless of genotype (Fig. 5A-H). However, significant differences in both OS (HR = 3.3, *P* = 0.0176) and RFS (HR = 3.1, *P* = 0.0277) were found in early-stage male patients with *KRAS* mutations albeit not in all male patients (Fig. 6A-D). In early-stage male patients with *KRAS* mutations, those with high pCASTOR1 scores, only 25% of them achieved 5-year OS (Fig. 6C) and none of them survived up to 5 years without experiencing an event of disease recurrence (Fig. 6D). These “pCASTOR1 high” early-stage male patients with *KRAS* mutations performed similarly to those of late-stage patients (Fig. 6C-D). For “pCASTOR1 low” early-stage male patients with *KRAS* mutations, 60% achieved both 5-year OS and RFS (Fig. 6C-D). Thus, high pCASTOR1 scores in the early-stage male patients with *KRAS* mutations conferred adverse disease outcomes with prognosis similar to those in the advanced stage cancer. In contrast, pCASTOR1 score failed to predict OS and RFS in early- or late-stage of male patients with *EGFR* mutations, or without any *KRAS* and *EGFR* mutations (Fig. 6E-H). It also failed to predict OS and RFS in early- or late-stage female patients regardless of the genotype (Fig. 7A-H).

## Discussion

By integrating signals of both nutrients and growth factors, CASTOR1 regulates mTORC1 activity [18–20]. As mTORC1 is commonly activated in cancer [18, 32], it is reasonable to speculate that pCASTOR1 can serve as a potential biomarker in cancer. Using a specific antibody, we examined pCASTOR1 level in tumor and non-tumor cells in LUAD tissues. We showed that pCASTOR1 scores were significantly higher in tumor cells than non-tumor cells irrespective of the presence of *KRAS* or *EGFR* mutations in tumor cells (Fig. 1C-F). However, there was no significant difference in pCASTOR1 level among different stages or grades of tumors (results not shown). These results are consistent with those of a previous report showing the lack of a correlation between tumor stage and mTORC1 activity in patients prior to chemotherapy [33]. Indeed, it has been reported that hyperactivation of mTORC1 activity occurs at the early stages of tumorigenesis in colorectal cancer and is involved in the transformation of normal cells to tumor cells [34, 35]. However, other studies have revealed that mTORC1 activity increases with tumor grade and is implicated in tumor invasive growth in colorectal cancer and colon adenocarcinoma [36, 37]. Our results suggest that pCASTOR1 change might be involved in the neoplastic transformation of cancer cells and its level might be maintained in subsequent tumor progression. This is consistent with the tumor suppressive function of CASTOR1, which must be inhibited in order to achieve constitutive activation of mTORC1 for tumor cell growth [21, 22].

In this study, we found that pCASTOR1 score was an independent and significant predictor of OS and RFS in male patients with *KRAS* mutations, and high pCASTOR1 scores predicted worse OS and RFS (Fig. 3C-D; Table 1). These results were not found in female patients with *KRAS* mutations (Fig. 4C-D, Table S3). Furthermore, we observed that high pCASTOR1 scores were predictive of worse OS and RFS in early-stage but not late-stage male LUAD patients with *KRAS* mutations (Fig. 6C-D).

LUAD is known to display sex-specific differences in frequency, response to treatment and patient survival. LUAD is the more common type of lung cancer in female patients [38], who also respond better to chemotherapy and have better survival than male patients [39, 40]. This could explain why pCASTOR1 scores were significantly and independently predictive of survival in male patients with *KRAS* mutations but not female patients (Figs. 3C-D and 4; Table 1). Additional studies are required to further determine the impact of sex and genotype on the prognostic value of pCASTOR1 in LUAD patients.

By further examining cancer stage, we have identified a prognostic value of pCASTOR1 level in early-stage LUAD male patients with *KRAS* mutations. Since *KRAS* mutations are known to constitutively activate the AKT/mTOR pathway through its effector proteins [41], it is reasonable to assume that the presence of a high pCASTOR1 score in LUAD with *KRAS* mutations is indicative of a dysregulated mTORC1 activity, and hence more aggressive tumor growth and worse patient survival. As a consequence, a high pCASTOR1 score was predictive of worse OS and RFS in early-stage male LUAD patients with *KRAS* mutations (Fig. 6C-D; Table 1). Furthermore, it has been reported that chemotherapy treatment of lung cancer with *KRAS* mutations causes hyperactivation of mTORC1 signaling [42]. Direct comparison of matched tissues before and after chemotherapy revealed that the mTOR activation markers, phosphorylated-mTOR and phosphorylated-S6 were upregulated in chemotherapy-treated LUAD with *KRAS* mutations but not in treatment-naïve tumors. In contrast, this phenomenon was not observed in matched tumors with a wild-type *KRAS* [42]. Importantly, treatment of lung cancer cells harboring *KRAS* mutations with the mTORC1 inhibitor rapamycin restored chemosensitivity of the resistant cells and, combination of rapamycin and chemotherapy showed strong synergistic effect in inhibiting tumor growth [42]. These results indicate that mTORC1 hyperactivation is a mechanism of chemoresistance of cancer cells with *KRAS* mutations [42]. Nevertheless, mTOR inhibitors failed in *KRAS* mutant NSCLC in other studies [43, 44]. In our current study, patients were mainly diagnosed and treated by chemotherapy, surgery and radiation therapy before the availability of targeted therapy or immunotherapy. Our results suggest that treatment of “pCASTOR1 high” early-stage male LUAD patients with *KRAS* mutations with the conventional approach might not be effective if they had hyperactivated mTORC1 activity prior to chemotherapy. Despite presenting with early-stage LUAD, these patients displayed survival outcomes similar to those presenting with an advanced stage LUAD. Hence, a different treatment approach might be needed to manage the disease in this subgroup of patients. In this case, pCASTOR1 could potentially serve as a biomarker to identify this subgroup of patients who might resist and fail to benefit from conventional treatment approaches, including chemotherapy.

Despite the availability of numerous approaches, treatment for early-stage NSCLC, especially stage I NSCLC is still largely confined to surgery, chemotherapy or radiation as a single or combined regimen. Stage II NSCLC patients, however, have slightly broader treatment options including immunotherapy or *EGFR*-mutant targeted therapy with Osimertinib [45, 46]. Other treatments including those targeting *KRAS*-G12C mutation (adagrasib or sotorasib) are mainly reserved for patients with an advanced-stage NSCLC [45]. NSCLCs with *KRAS* mutations can be good candidates for immunotherapy because of the high tumor mutation burden [47]. In fact, tumors with both *KRAS* and *TP53* dual mutations exhibit a high rate of PD-L1 positivity and are more sensitive to PD-1 inhibitors [48]. In line with the current practice, we propose that “high pCASTOR1” male early-stage LUAD patients with *KRAS* mutations should be considered for approaches other than chemotherapy alone as the first-line treatment including immunotherapy or targeted therapies.

While this study revealed pCASTOR1 as a potential biomarker for male early-stage LUAD patients with *KRAS* mutations, we acknowledge that the major limitation of this study being the small sample size in each genotype subgroup of both male and female patients, with each of them having less than 50 patients. Nevertheless, the findings in this study still maintain its robustness in both the univariate and multivariate analyses despite the small sample size.

## Conclusion

In summary, we have developed a specific antibody for pCASTOR1 and revealed higher pCASTOR1 levels in tumor than non-tumor cells. We have shown that pCASTOR1 score is a prognostic marker in male LUAD patients with *KRAS* mutations. Early identification of this subgroup of patients could help tailor specific treatments to improve their survival outcomes.

## Electronic supplementary material

Below is the link to the electronic supplementary material.

Supplementary Material 1

Supplementary Material 2

## Data Availability

All data generated or analyzed during this study are included in this manuscript and its supplementary information files.
