# Supplementary figures and images for: CASTOR1 phosphorylation predicts poor survival in male patients with KRAS-mutated lung adenocarcinoma
Source: Cell Biosci. 2024 Oct 9;14:127. doi: 10.1186/s13578-024-01307-4 (PMC11465729; doi:10.1186/s13578-024-01307-4)

### Figure 1A

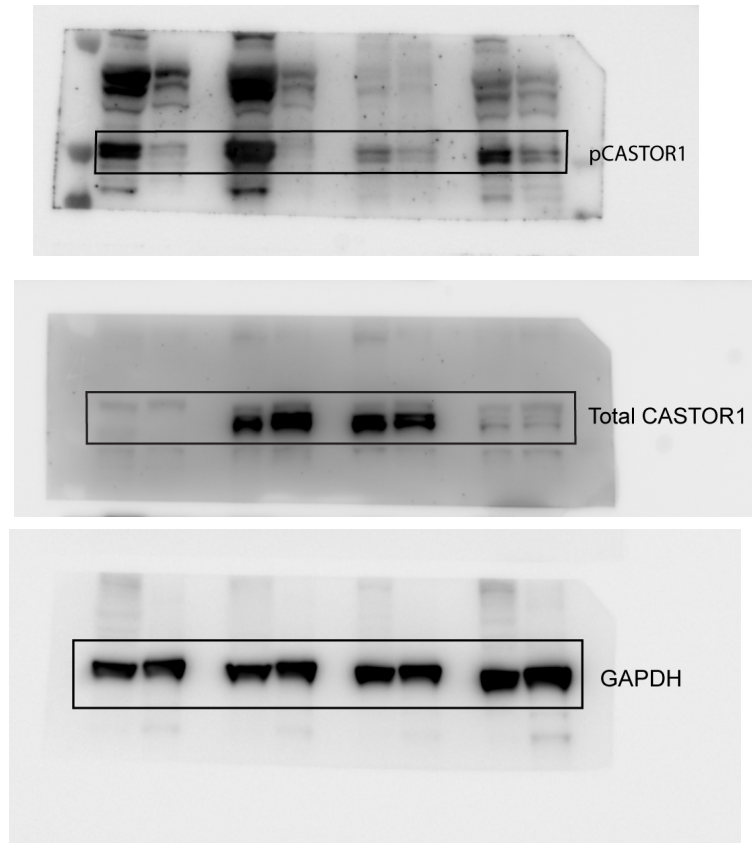

Supplement: Supplementary file 1 — Supplementary Material 1 [file 13578_2024_1307_MOESM1_ESM.pdf]
