# Supplementary material for: CASTOR1 phosphorylation predicts poor survival in male patients with KRAS-mutated lung adenocarcinoma
Source: Cell Biosci. 2024 Oct 9;14:127. doi: 10.1186/s13578-024-01307-4 (PMC11465729; doi:10.1186/s13578-024-01307-4)
Supplement: Supplementary file 2 — Supplementary Material 2 [file 13578_2024_1307_MOESM2_ESM.docx]

**Supplementary Information**

**CASTOR1 Phosphorylation Predicts Poor Survival in Male Patients with *KRAS*-Mutated Lung Adenocarcinoma**

Suet Kee Loo^1,2^, Gabriel Sica^3,4^, Xian Wang^1,2^, Tingting Li^1,2^, Luping Chen^1,2^, Autumn Gaither Davis^5^, Yufei Huang^1,5,6^, Timothy F. Burns^5,7,8^, Laura P. Stabile^7,8^, Shou-Jiang Gao^1,2^*

*Correspondence:

Shou-Jiang Gao

gaos8@upmc.edu

^1^Cancer Virology Program, UPMC Hillman Cancer Center, Pittsburgh, Pennsylvania, USA

^2^Department of Microbiology and Molecular Genetics, University of Pittsburgh School of Medicine, Pittsburgh, Pennsylvania, USA

^3^Department of Pathology, University of Pittsburgh School of Medicine, Pittsburgh, Pennsylvania, USA

^4^UPMC Presbyterian Hospital, University of Pittsburgh Medical Center, Pittsburgh, Pennsylvania, USA

^5^Department of Medicine, University of Pittsburgh School of Medicine, Pittsburgh, PA, USA

^6^Department of Electrical and Computer Engineering, Swanson School of Engineering, University of Pittsburgh, Pittsburgh, PA, USA

^7^Cancer Biology Program, UPMC Hillman Cancer Center, Pittsburgh, Pennsylvania, USA

^8^Department of Pharmacology and Chemical Biology, University of Pittsburgh School of Medicine, Pittsburgh, Pennsylvania, USA

**
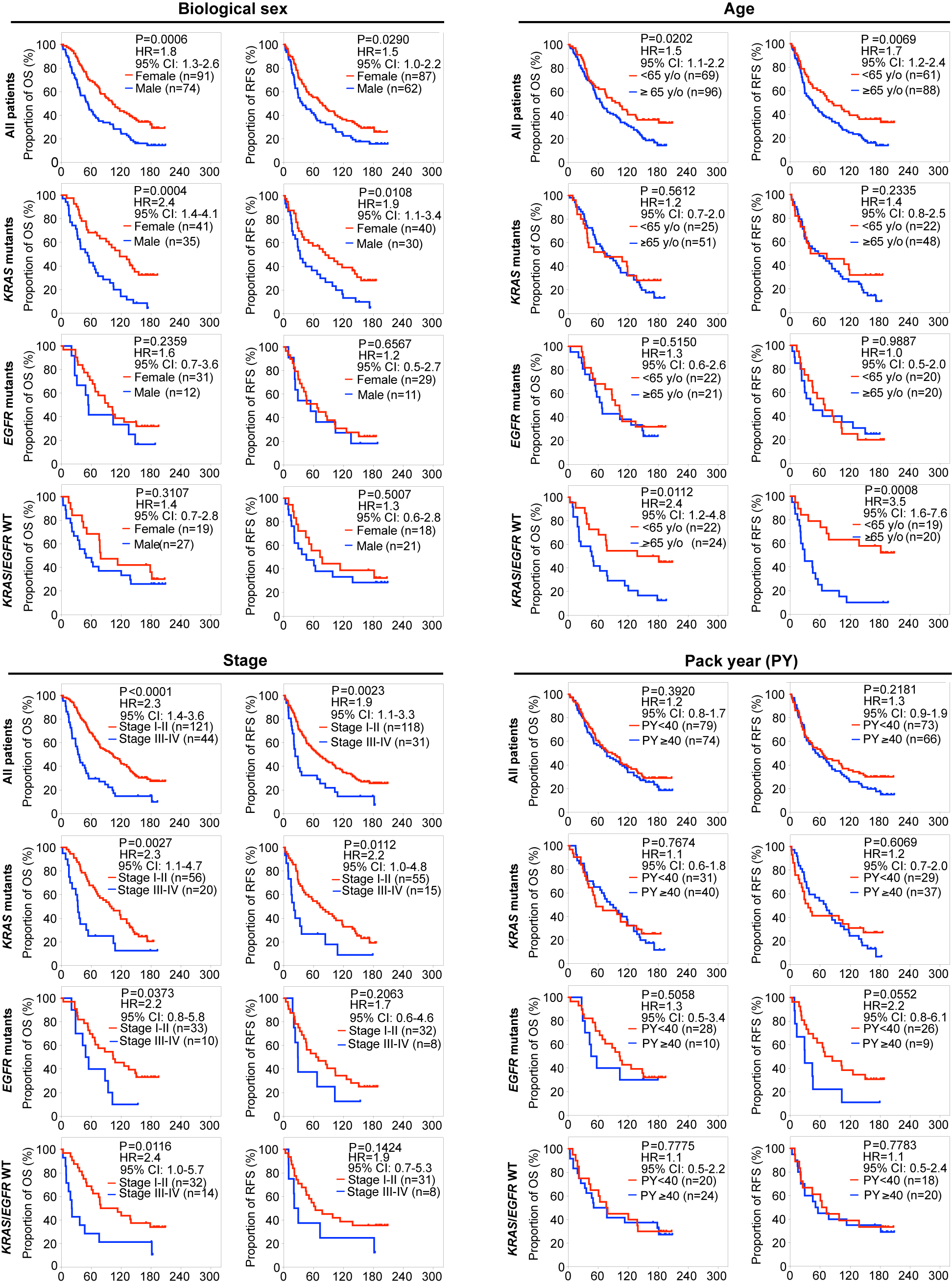
**

**Fig. S1.** OS and RFS of all LUAD patients, patients with *KRAS* mutations (*KRAS* mutants), *EGFR* mutations (*EGFR* mutants), or without any *KRAS* and EGFR mutations (*KRAS*/*EGFR* WT), stratified according to biological sex, age at diagnosis, cancer stage and smoking pack year. X-axis: months elapsed. Y-axis: proportion of OS (%) or RFS (%). HR: Hazard ratio; CI: Confidence interval; y/o: year-old. Pack year (PY): Total cigarette packs smoked in lifetime.

| **Table S1** Clinical and demographic characteristics of LUAD patients | |
| --- | --- |
| Subject (n) | 165 |
| Age (years old) |  |
| Median | 66.0 |
| Range | 39.0-88.0 |
| Sex (%) |  |
| Male | 74 (44.8) |
| Female | 91 (55.2) |
| Stage (%) |  |
| I-II | 121 (73.3) |
| III-IV | 44 (26.7) |
| Race (%) |  |
| White | 153 (92.7) |
| Black | 10 (6.1) |
| Others | 2 (1.2) |
| Smoking status (%) |  |
| Never | 22 (13.3) |
| Former | 101 (61.2) |
| Current | 42 (25.5) |
| Pack year |  |
| <40 | 79 (51.6) |
| ≥40 | 74 (48.4) |
| *KRAS* mutation (%) |  |
| Yes | 76 (46.1) |
| No | 89 (53.9) |
| *EGFR* mutation (%) |  |
| Yes | 43 (26.1) |
| No | 122 (73.9) |
| Without *KRAS* or *EGFR* mutation (%) |  |
| Yes | 46 (27.9) |
| No | 119 (72.1) |

Pack year: Total cigarette packs smoked in lifetime.

**Table S2** Multivariate analysis of all LUAD patients

| Characteristics | OS | | | |  | RFS | | | |
| --- | --- | --- | --- | --- | --- | --- | --- | --- | --- |
|  | n (%) | HR | 95.0% CI | P-value |  | n (%) | HR | 95.0% CI | P-value |
| Age (year-old) |  |  |  |  |  |  |  |  |  |
| <65 | 63 (41.2) | 1.0 |  |  |  | 56 (40.3) | 1.0 |  |  |
| ≥65 | 90 (58.8) | 1.5 | 1.0-2.2 | 0.0479 |  | 83 (59.7) | 1.8 | 1.2-2.7 | 0.0046 |
| Sex |  |  |  |  |  |  |  |  |  |
| Female | 84 (54.9) | 1.0 |  |  |  | 80 (57.6) | 1.0 |  |  |
| Male | 69 (45.1) | 1.8 | 1.2-2.6 | 0.0024 |  | 59 (42.4) | 1.6 | 1.1-2.3 | 0.0257 |
| Stage |  |  |  |  |  |  |  |  |  |
| I & II | 113 (73.9) | 1.0 |  |  |  | 110 (79.1) | 1.0 |  |  |
| III & IV | 40 (26.1) | 2.5 | 1.7-3.8 | <0.0001 |  | 29 (20.9) | 2.6 | 1.6-4.0 | <0.0001 |
| Pack year |  |  |  |  |  |  |  |  |  |
| <40 | 79 (51.6) | 1.0 |  |  |  | 73 (52.5) | 1.0 |  |  |
| ≥40 | 74 (48.4) | 1.2 | 0.8-1.7 | 0.4395 |  | 66 (47.5) | 1.2 | 0.8-1.8 | 0.2672 |
| pCASTOR1 |  |  |  |  |  |  |  |  |  |
| Low | 112 (73.2) | 1.0 |  |  |  | 101 (72.7) | 1.0 |  |  |
| High | 41 (26.8) | 1.0 | 0.7-1.6 | 0.9191 |  | 38 (27.3) | 0.9 | 0.6-1.4 | 0.5942 |
|  |  |  |  |  |  |  |  |  |  |

HR: hazard ratio; CI: Confidence interval; OS: Overall survival; RFS: Recurrence-free survival; Pack year: Total cigarette packs smoked in lifetime.

**Table S3** Multivariate analysis of female patients with *KRAS* mutations

| Characteristics | OS | | | |  | RFS | | | |
| --- | --- | --- | --- | --- | --- | --- | --- | --- | --- |
|  | n (%) | HR | 95.0% CI | P-value |  | n (%) | HR | 95.0% CI | P-value |
| Age (year-old) |  |  |  |  |  |  |  |  |  |
| <65 | 13 (34.2) | 1.0 |  |  |  | 13 (35.1) | 1.0 |  |  |
| ≥65 | 25 (65.8) | 0.8 | 0.4-1.9 | 0.6715 |  | 24 (64.9) | 1.0 | 0.4-2.3 | 0.9573 |
| Stage |  |  |  |  |  |  |  |  |  |
| I & II | 31 (81.6) | 1.0 |  |  |  | 31 (83.8) | 1.0 |  |  |
| III & IV | 7 (18.4) | 3.0 | 1.1-8.3 | 0.0350 |  | 6 (16.2) | 3.4 | 1.2-9.7 | 0.0216 |
| Pack year |  |  |  |  |  |  |  |  |  |
| <40 | 18 (47.3) | 1.0 |  |  |  | 17 (45.9) | 1.0 |  |  |
| ≥40 | 20 (52.6) | 0.8 | 0.4-1.7 | 0.5193 |  | 20 (54.1) | 1.0 | 0.5-2.2 | 0.9922 |
| pCASTOR1 |  |  |  |  |  |  |  |  |  |
| Low | 27 (71.1) | 1.0 |  |  |  | 26 (70.3) | 1.0 |  |  |
| High | 11 (28.9) | 1.7 | 0.7-4.1 | 0.2727 |  | 11 (29.7) | 1.2 | 0.5-2.9 | 0.6755 |
|  |  |  |  |  |  |  |  |  |  |

HR: hazard ratio; CI: Confidence interval; OS: Overall survival; RFS: Recurrence-free survival; Pack year: Total cigarette packs smoked in lifetime.
